# The Antitoxin Treatment of Diphtheria

**Published:** 1901-02-16

**Authors:** 


### THE ANTITOXIN TREATMENT OF DIPHTHERIA.

After six years' experience with the antitoxin treatment of diphtheria, Dr. Henry Koester,[1] of New York, sums up his conclusions. He says that antitoxin, to obtain its best effects, must be used fearlessly and in quantity sufficient to be effective, which is never less than 2,000 units, and that even in very mild cases, and it must be used at once. In severe cases at least 3,000 units must be used, and this dose must be repeated in from twelve to twenty-four hours. Antitoxin itself, he says, contrary to the expressed belief of some observers, never does any harm. "When called to see a child sick with an ailment which may or may not be diphtheria, the first step in examination is to look at the throat. If any suspicion of exudation is present, an injection of antitoxin should be the next move, after which a culture should be taken. If a subsequent examination proves the case to be one of diphtheria, the patient has been put in the best possible condition to combat the disease; if the bacteriological test proves that you were mistaken, no harm has been done." "One of the most important facts to bear in mind is," he says, "that the dose of antitoxin is to be regulated, not by the age of the patient, but by the severity of the disease and the length of time he has been ill." There may be some exceptions, but it is a safe rule to follow, whether we meet with a child of one year or five years; and even when the age of the patient is more than five years, it will not be necessary to increase the dose very much. Except in cases under a month old, 2,000 units is the smallest dose; and when the disease has invaded the uvula and pharynx, 3,000 to 4,000 units should be the initial dose, and more still if the nares are affected or if the case is one of diphtheritic croup. In the latter case we ought always to be prepared to intubate at a moment's notice. It is not an uncommon experience, he says, to find in a family in which one child has diphtheria other children who are apparently not ill, but have colds in the head. One child may have a running nose, another a muco-purulent discharge from the eyes; and a bacteriological examination will reveal the presence of the diphtheria bacillus. In these children a single injection of 2,000 units of antitoxin will produce marvellous results. As a rule this single injection will stop the discharge without further trouble. Dr. Koester prefers to give the injection in the gluteal region. "If the assistant places the child over one knee and with his free leg over the patient's, it is very easy to hold the child firmly, and the gluteal muscles offer a convenient place for operation." The needle must be carried deep into the muscle. One of the most valuable features of the antitoxin treatment of diphtheria is its use in immunisation. In large families in tenement houses where isolation of the afflicted person is impossible unless removed to hospitals, an injection of antitoxin into those not affected will prevent the spread of the disease. The reports of physicians in institutions in which children are kept seems to fix the immunising dose at 150 to 300 units, but Dr. Koester says that in tenement- and flat-house districts, where children cannot be closely watched, and where isolation cannot be practised, it is his universal custom to give from 300 to 500 units as a prophylactic dose. From an experience of nearly 1,800 cases in tenement-house practice he says that in only four of these cases was a membrane produced within twelve to thirty days after the injection. "Never have I seen any complications or sequelæ follow the use of antitoxin as an immunising agent, excepting the occurrence of an urticaria-like rash." Dr. Koester goes on then to describe the other measures which may have to be adopted in the treatment of diphtheria, but to us the chief interest lies in what he says concerning the efficiency of antitoxin if given early enough, and the dosage in which it must be used, in regard to both of which he is strongly supported by those on this side the water who have had most to do with the practical application of the antitoxin treatment. It seems to us high time that we should make up our minds much more definitely and practically than we seem hitherto to have done on this important subject. If it is really the fact that by the use of antitoxin as a prophylactic in home practice it is possible to prevent the spread of the disease in those cases in which it is impossible to secure proper isolation, it seems clear that it should be made the duty of the sanitary authorities to furnish a much more plentiful supply of antitoxin than is to be got at the present time, and that it should be rendered available much more readily than is now the case. We do not say that the production of a temporary immunity by injection is an ideal way of dealing with an outbreak, and we look upon the New York arrangements in this regard with a certain degree of wonder; but if Dr. Koester's conclusions are correct, it ought to be quite possible in country districts, where the lack of hospital accommodation is often more felt than in large towns, to stamp out isolated outbreaks of diphtheria quickly and safely.

Dr. Koester's conclusions are (1) that antitoxin is a positive cure for diphtheria when employed in sufficient quantity and sufficiently early in the disease; (2) that even when employed too late to produce its specific action it cannot do harm; and (3) that when used before the invasion of diphtheria, antitoxin possesses a positive immunising power which lasts about thirty days.

[1] New York Medical News, Jan. 19.

